# Genome-Wide Analysis Reveals Changes in Polled Yak Long Non-coding RNAs in Skeletal Muscle Development

**DOI:** 10.3389/fgene.2020.00365

**Published:** 2020-04-15

**Authors:** Xiaoming Ma, Donghai Fu, Min Chu, Xuezhi Ding, Xiaoyun Wu, Xian Guo, Qudratullah Kalwar, Jie Pei, Pengjia Bao, Chunnian Liang, Ping Yan

**Affiliations:** ^1^Animal Science Department, Lanzhou Institute of Husbandry and Pharmaceutical Sciences, Chinese Academy of Agricultural Sciences, Lanzhou, China; ^2^Key Laboratory for Yak Genetics, Breeding, and Reproduction Engineering of Gansu Province, Gansu Provincial Key Laboratory of Yak Breeding Engineering, Lanzhou Institute of Animal Husbandry and Veterinary Medicine, Chinese Academy of Agricultural Sciences, Lanzhou, China

**Keywords:** lncRNA, RNA-sequencing, polled yak, longissimus dorsi, muscle development

## Abstract

Long non-coding RNAs (lncRNAs) have been extensively studied in recent years. Numerous lncRNAs have been identified in mice, rats, and humans, some of which play important roles in muscle formation and development. However, little is known about lncRNA regulators that affect muscle development in yak (*Bos grunniens*). LncRNA expression during skeletal muscle development in yak was analyzed by RNA sequencing at three development stages: 3 years (group A), 6 months (group M), and 90-day-old fetuses (group E). A total of 1180 lncRNAs were identified in the three development stages. Compared with group E, 154 were upregulated and 130 were downregulated in group A. Compared with group A, 31 were upregulated and 29 were downregulated in group M. Compared with group E, 147 were upregulated and 149 were downregulated in group M (padj < 0.001, |log2FC| > 1.2). In addition, functional annotation analysis based on gene ontology (GO) and the Kyoto protocol encyclopedia of genes and genomes (KEGG) database showed that differentially expressed lncRNAs (DElncRNAs) were cis–trans target genes. The results showed that DElncRNAs were mainly involved in PI3K-Akt signaling pathway, focal adhesion, MAPK signaling pathway, apoptosis, and p53 signaling pathway. Furthermore, RTL1, IGF2, MEF2C, Pax7, and other well-known muscle development regulators were included in a co-expression network of differentially expressed target genes and lncRNAs. These data will help to further clarify the function of lncRNAs in the different stages of skeletal muscle developmental in yak.

## Introduction

The yak (*Bos grunniens*) is a unique mammal that inhabits the plateau, with 14 million animals worldwide (Wiener et al., 2011; Qiu et al., 2012), distributed at high altitudes of 3000–5000 m. Yak were domesticated by humans between 7000 and 10,000 years ago (Qi et al., 2013) and have since migrated along with the Tibetan population throughout the plateau, providing local residents with meat, milk, transportation, shelter, and fuel (Wang et al., 2018). The polled yak, known as the “Datong Yak,” is a new breed developed by the Lanzhou Institute of Animal Husbandry and Veterinary Medicine at the Chinese Academy of Agricultural Sciences. Compared with other yak breeds, these animals have a gentle and calm temperament, reducing the natural injury rate. Polled yak are also easy to raise and manage, which is beneficial for increasing the stock density and facilitating the feeding. Thus, the characteristics of polled yak are of great significance for large-scale intensive standardized farming in the Qinghai-Tibet Plateau.

Long non-coding RNAs (lncRNAs) are a class of transcripts greater than 200 nucleotides (nt) in length. Due to the lack of typical open reading frames (ORFs), it was previously thought that lncRNAs were not translated into proteins (Mattick and Rinn, 2015); however, recent studies have shown the presence of short (or small) ORFs (sORFs) that encode small peptides that have important biological functions (Anderson et al., 2015, 2016; Nelson et al., 2016). These transcripts were commonly regarded as “junk RNA”; however, with recent advances in sequencing technology, lncRNAs have emerged as key regulators of gene expression. LncRNAs are involved in many biological processes such as development (Paralkar et al., 2014; Zhao et al., 2015), transcriptional regulation (Vance and Ponting, 2014), and cell differentiation (Cesana et al., 2011).

Numerous lncRNAs have been discovered in livestock such as *Bos taurus* (Billerey et al., 2014; Koufariotis et al., 2015), *Sus scrofa* (Ran et al., 2016; Yang et al., 2019), and *Gallus gallus* (Ouyang et al., 2017). Recent studies have suggested that lncRNAs are involved in the growth of skeletal muscle, and related research has been carried out in livestock (Li et al., 2012; Wang et al., 2015); however, the functions lncRNAs in yak skeletal muscle at various stages of development remain to be elucidated. Therefore, we studied the three key stages lncRNAs in the expression pattern and the potential role of lncRNAs in yak muscle at three developmental stages (fetus: 90 day; juvenile: 6 months; and adult: 3 years). Our study will provide a useful resource that provides an improved understanding of the regulatory functions of lncRNA in yak and annotated yak genomes, as well as contributing to a better understanding of mammalian skeletal muscle development.

## Materials and Methods

### Animals

All yaks were handled in strict accordance with the Animal Ethics Procedures and Guidelines of the People’s Republic of China. The present study was approved by the Animal Administration and Ethics Committee of Lanzhou Institute of Husbandry and Pharmaceutical Sciences of Chinese Academy of Agricultural Sciences (Permit No. 2019-002).

### Tissue Collection

Polled yaks were selected from livestock in the Ashidan Mountain area of Qinghai Province. All yaks were raised using the same feeding and management practices at the same farm. A total of nine healthy female yak were selected covering three age groups: 6 months old (group M), 3 years old (group A), and 90-day fetuses (group E). Fetal age was estimated from the crown rump length (Richardson et al., 1990). The animal samples (longissimus dorsir muscle) used in this study were obtained after slaughter. Samples were divided into 0.5 cm^3^ portions, treated with RNAlater (Qiagen, Hilden, Germany) and stored overnight at 4°C before storage in liquid nitrogen for RNA extraction.

### Clustering, Sequencing, and Transcriptome Assembly

Total RNA was extracted from the longissimus dorsi muscle tissues using TRIzol regent (Invitrogen, Carlsbad, CA, United States) and the RNA quality was checked. RNA integrity was evaluated by 1% agarose gel electrophoresis and total RNA concentration and purity were determined by Agilent 2100 assay, RIN, and 28S/18S or 23S/16S analysis. Then TruSeq Stranded Total RNA with ribo-zero Gold kit (Illumina, San Diego, CA, United States) was used for common animals. Genomic DNA was removed from the sample using DNase I (Fermentas, Vilnius, Lithuania) before cDNA was generated from 2 μg of total RNA in nine samples using the Illumina HiSeq^TM^ 2500 system (Illumina Corp., San Diego, CA, United States) according to the manufacturer’s instructions. After quantitative analysis using an Agilent 2100 Bioanalyzer (Agilent, Santa Clara, CA, United States), specific strand libraries were sequenced on an Illumina HiSeq^TM^ 2500 instrument to generate 150-nt paired-end sequences. Libraries were constructed and sequenced using the Illumina platform by OE Biotech Co. (Shanghai, China).

The raw Illumina sequencing data were then quality-checked using FASTQC tools (Andrews, 2010). At the 3′-end, the quality of each substrate was significantly reduced and trimmed with sliding windows using Trimmomatic; once cut, the average base quality drops to within 10% of Phred score (90% accuracy) (Bolger et al., 2014). The quality of the trimmed reads was rechecked with the FASTQC tool, and HISAT2 was used to align the remaining high-quality clean reads with the yak genome (Kim et al., 2015). A mapping read segment for each sample was assembled using StringTie (v1.3.1) following a reference-based approach (Pertea et al., 2015). Finally, the compiled transcript was annotated by the Cuffcompare program in the Cufflinks package.

### Identification of lncRNAs

**FIGURE 1:**
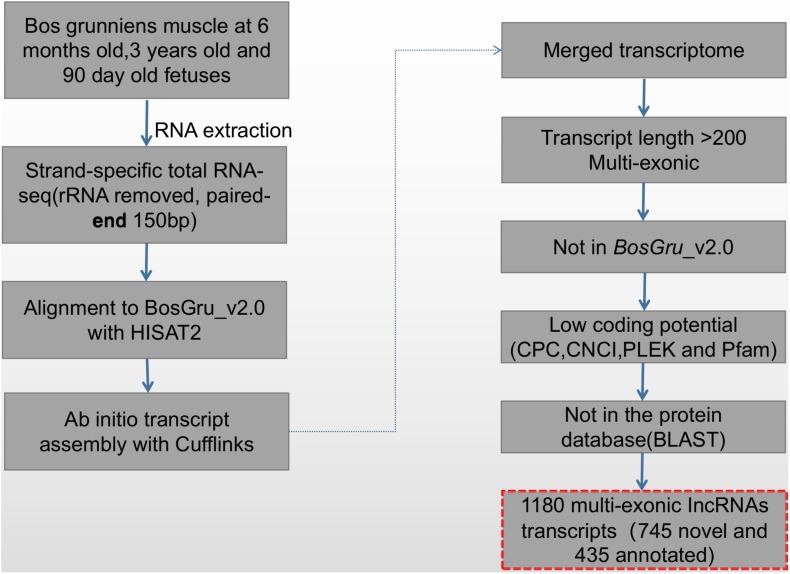
The pipeline used to identify novel lncRNAs.

The screening and identification process of lncRNA was carried out according to the protocol summarized in Figure 1. Transcript exon number screening was conducted by filtering the low expression levels in splicing results and low-confidence single exon transcripts; transcripts with a number of exons ≥ 2 were selected. A selection of transcripts with a transcript length of ≥ 200 nt were then selected before screening of the annotated transcripts. The transcripts overlapping the exon region of the database annotation were screened, and lncRNAs in the database of annotated ncRNAs were selected for subsequent analysis. For screening of transcript expression levels, the expression level of the master transcript was calculated, and transcripts with FPKM ≥ 0.5 (screening threshold for single exon transcript 2) were selected. The coding potential screening was predicted according to the Coding-Non-Coding Index (CNCI) (Sun et al., 2013), Coding Potential Calculator (CPC) (Kong et al., 2007), and PFAM (pfamscan) (Bateman et al., 2004). Only transcripts that were simultaneously considered to have no coding potential by the four software programs were identified as lncRNAs. The PLEK (Li et al., 2014) tool was used to distinguish lncRNA from mRNA based on an optimized k-mer strategy and support vector machine algorithm. The accuracy of this method in the prediction of human lncRNA is as high as 95.6%. Transcripts predicted to have coding potential were identified as novel mRNAs.

### Principal Component Analysis (PCA) and the Analysis of Differential Expression of lncRNA

Correlation analysis was performed on the lncRNA expression levels of nine samples by principal component analysis (PCA) (Wickham, 2016). In addition, the FPKM values of lncRNA in each library were calculated using cuffdiff software (Trapnell et al., 2012). Differentially expressed lncRNA (DElncRNA) between any two libraries were identified by DEseq (Anders and Huber, 2012). Padj-values < 0.001 and |log 2 (fold change)| > 1.2 were used as threshold values to evaluate the statistical significance of the differences in lncRNA levels. The Heatmap software package in R was used to systematically analyze all DElncRNA clusters in the nine libraries^1^.

### LncRNA Target Gene Prediction

LncRNAs regulate the expression level of protein-coding genes predominantly in the cis or trans modes (Mercer et al., 2009). The cis mode of action is also known as position-dependent target gene regulation. Based on the positional relationship between lncRNA and mRNA, lncRNAs regulate the transcriptional activation of protein-coding genes within 10 K/100 K of its adjacent position to affect its expression level. Trans regulation refers to the ability of lncRNA to regulate the expression of a protein-coding gene with which it shares an expression-related gene. Protein-coding genes correlating with lncRNA expression were screened based on the Pearson correlation coefficient (Irl > 0.95). The prediction of lncRNA function is based first on prediction of the protein-coding target gene regulated by the two modes (cis or trans), followed by the functional enrichment analysis of the target gene (Guil and Esteller, 2012).

### Enrichment Analysis of Differentially Expressed lncRNAs

Functional annotation of lncRNAs and mRNAs was performed the based on gene ontology (GO) and the Kyoto protocol encyclopedia of genes and genomes (KEGG) database^2^ using the clusterprofiles R package (Yu et al., 2012) and KOBAS v2.0 software (Xie et al., 2011), respectively. *P* < 0.05 was considered to indicate statistically significant enrichment.

### Construction of lncRNA and mRNA Networks

The interactions between target genes and differentially expressed genes in the development of yak muscle development were evaluated based on the targeting relationships between mRNAs and lncRNAs. The GO and KEGG classification keywords were used to identify the muscle growth and development network and the lncRNA–mRNA interaction network was constructed using Cytoscape open software platform (V3.1.1) (Saito et al., 2012).

### Gene Expression Validation by qRT-PCR

Total RNA from muscle tissue samples was prepared as described in Section “Clustering, Sequencing, and Transcriptome Assembly.” The samples were diluted to 1 μg/ml with DEPC-treated double distilled water and first strand cDNA was prepared using the PrimeScript^TM^ first strand cDNA Synthesis Kit (TaKaRa). We selected five DElncRNAs and their targeted DEGs; the lncRNAs (CREB5, ITGB3, TCONS_00007120, IGF2, TCONS_00024310, RTL1, XR_314844.1, PAX7, TCONS_00034044, MEF2C, TCONS_00027657, TCONS_00014143, TCONS_00027958, and XR_001351404.1) were involved in the process of muscle growth. The relative expression of 15 candidate differentially expressed genes/lncRNAs in the nine muscle tissue samples was analyzed by qRT-PCR the using the cDNA as a template and SYBR^®^Premix Ex Taq^TM^ III Reagent Gold (TaKaRa) on the CFX Connect^TM^ Real Time PCR Detection System. Details of the primers designed to amplify the 15 candidate differentially expressed genes/lncRNAs are shown in Supplementary Table S1; the β-actin gene as the internal reference. The mean number of cycles required to pass the fluorescence threshold (Ct value) of each sample was used to calculate relative gene expression using the 2^–ΔΔCt^ method. The reliability of the sequencing results was evaluated by correlation analysis of the RT-qPCR and RNA-Seq data.

## Results

### Overview of the Sequencing Data

To identify the lncRNAs and mRNAs expressed during the development of the longissimus longus muscle, we constructed nine cDNA libraries. E6, E7, and E8 served as replicate libraries for group E (90-day fetuses). M2, M3, and M4 served as replicate libraries for group M (6 months old). A6, A7, and A10 served as replicate libraries for group A (3 years old). Three biological duplicates were prepared for each stage of development. A total of 887.98 M raw reads were obtained from all nine libraries. After removing the low-quality reads and adapter fragments, 850.38 M clean reads remained, corresponding to 93.41% of the total raw reads in the library. The Q30% values in all the libraries exceeded 95%, indicating the high quality of the clean reads. In total, 95.18–96.83% of the clean reads from all the libraries were successfully located in the reference genome. Of the successfully mapped reads, 88.81% of the uniquely mapped reads were used for transcript construction (Supplementary Table S2).

### Identified lncRNAs

**FIGURE 2:**
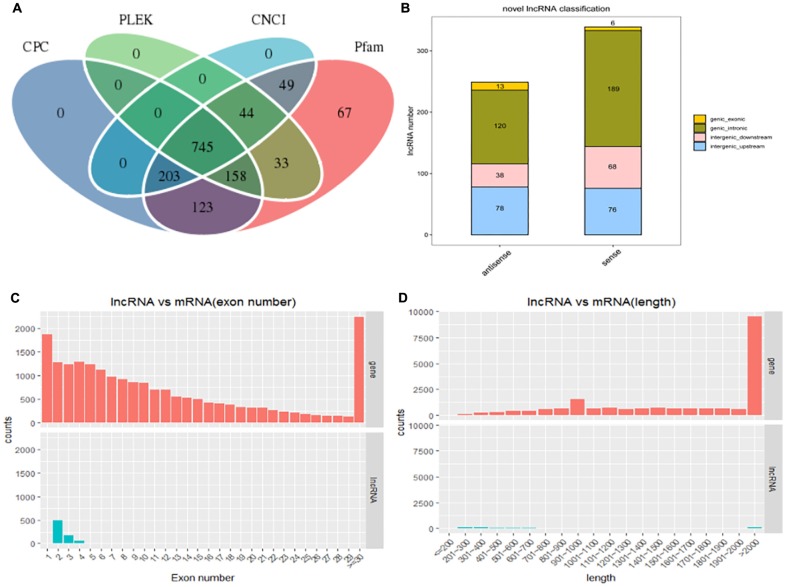
The long non-coding RNA (lncRNA) characteristic of yak skeletal muscle. **(A)** Venn diagram showing lncRNA transcripts from four datasets (CPC, CNCI, Pfam, and PLEK). **(B)** The positional relationship between lncRNA and known protein-encoded transcripts counting lncRNA types from four aspects: direction, type, location, and subtype. **(C)** The length distribution of lncRNAs and mRNAs. **(D)** The number of exons per lncRNA and mRNAs.

After filtering and coding capacity evaluation steps, a total of 1180 lncRNA (435 known and 745 novel) (Figure 2A) library transcripts were generated and expressed in muscle tissue for further analysis. In addition, a total of 20,506 mRNAs were identified. LncRNA genes encoding near as were used as the lncRNA genome background to provide an in-depth understanding of the functions of lncRNAs during yak muscle development. The following lncRNAs were identified: six exonic sense, 189 intronic sense, 68 intergenic downstream sense, 76 intergenic upstream sense, 13 exonic antisense, 120 intronic antisense, and 38 intergenic downstream antisense (Figure 2B and Supplementary Table S3). Because the yak reference genomic data are not complete, nearby coding genes were not identified for some of the lncRNAs. LncRNAs with a length greater than 2000 bp represented the highest proportion (Figure 2C) and most lncRNAs contained two exons (Figure 2D). Overall, the distribution of lncRNA and mRNA lengths was consistent, and the transcript lengths of lncRNAs were longer than those of the mRNAs (Figures 2C,D).

### PCA and Identification of Differentially Expressed lncRNAs

**FIGURE 3:**
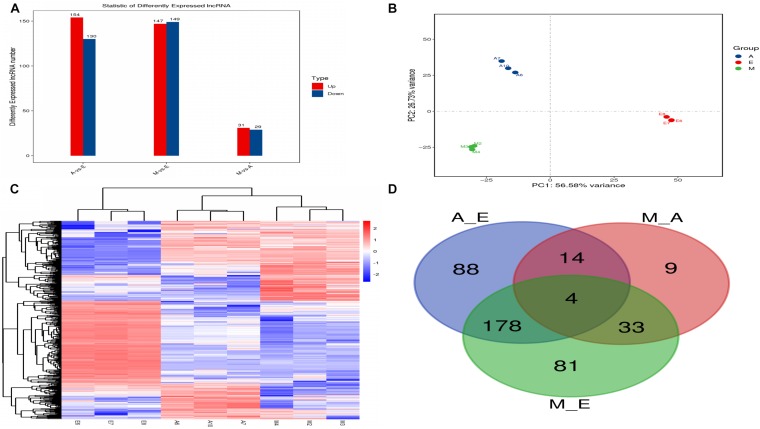
The number of differentially expressed lncRNAs in (control/experiment) A/E, M/A, and M/E comparisons. **(A)** Total number of differentially expressed lncRNAs in each comparison. **(B)** Principal component analysis of nine muscle samples. **(C)** The hierarchical clustering of differentially expressed lncRNAs. **(D)** Venn diagram showing the distribution of differentially expressed lncRNAs.

The expression level of each lncRNA transcript was estimated using cuffdiff software. In this study, we identified a total of 407 DElncRNA transcripts (Figure 3A and Supplementary Table S4). Compared with group E, 154 DElncRNAs were upregulated and 130 were downregulated lncRNAs in group A. Compared with group A, 31 DElncRNAs were upregulated and 29 were downregulated in group M. Compared with group E, 147 DElncRNAs were upregulated and 149 were downregulated in group M. Clustering heat maps and PCA were used to evaluate the expression patterns of DElncRNAs and to explore the relationships between the libraries. The results obtained from analysis of biological replicate samples at each growth stage were found to be clustered together (Figures 3B,C). As shown in Figure 3D, differentially expressed genes were common among the three comparisons (four lncRNAs).

### LncRNA Target Genes and Functional Analysis

The gene transcribed in the l00 kb down/upstream window is generally considered to be a cis target gene, while trans target genes are predicted by calculating the correlation with the expression of lncRNAs (correlation coefficient ≥ 0.95). Based on this approach, we selected the top 200 target genes for subsequent functional cluster analysis.

**FIGURE 4:**
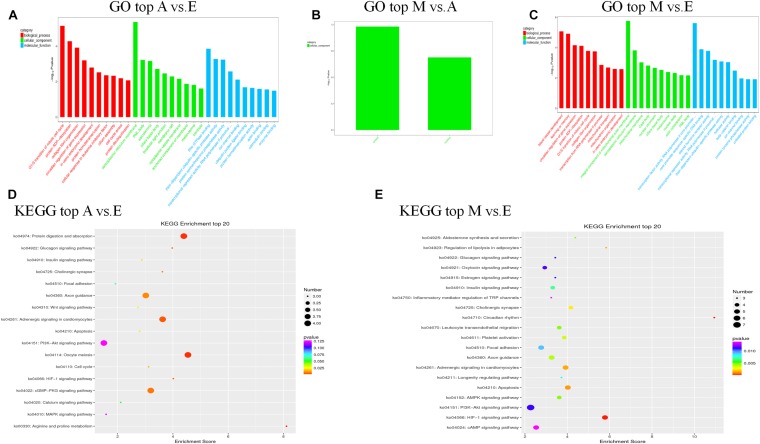
The top GO and KEGG enrichment analyses of the cis-target genes of the differentially expressed lncRNAs. **(A–C)** The top GO terms enriched by the target genes of the differentially expressed lncRNAs in each comparison. **(D,E)** The top KEGG terms enriched by differentially expressed lncRNAs in each comparison (A–E, M–E).

First, we predicted the biological function of target genes involved in cis regulation that were located within 100 K upstream and downstream of differentially expressed lncANA. In the A vs. E group, 138 lncRNAs (106 annotated lncRNAs and 32 novel lncRNAs) corresponded to 82 target genes. DEIncRNA target genes were enriched in 99 GO functions, of which only 39 were significantly enriched (*P* < 0.05). The results showed that these lncRNA targets were enriched in multiple processes, such as sarcoplasmic reticulum membrane, G1/S transition of mitotic cell cycle, actin cytoskeleton organization, and *in utero* embryonic development (Figures 4A,D and Supplementary Table S5). KEGG analysis of these DElncRNAs indicated that target genes were involved protein digestion and absorption, HIF-1 signaling pathway, glucagon signaling pathway, and insulin signaling pathway. In the M vs. A group, 12 lncRNAs (10 annotated lncRNAs and two novel lncRNAs) corresponded to 10 target genes. DEIncRNA target genes were enriched in two GO functions, of which none were significantly enriched terms (*P* > 0.05) (Figure 4B and Supplementary Table S5). In the M vs. E group, 153 lncRNAs (118 annotated lncRNAs and 35 novel lncRNAs) corresponded to 205 target genes. DEIncRNA target genes were enriched in 141 GO functions, of which only 64 were significantly enriched (*P* < 0.05). The *in utero* embryonic development, muscle organ development, and pathways are related to muscle development and energy metabolism. KEGG analysis of these DElncRNAs indicated that target genes were involved in the HIF-1 signaling pathway, apoptosis, AMPK signaling pathway, insulin signaling pathway, focal adhesion, and PI3K-Akt signaling pathway (Figures 4C,E and Supplementary Table S5).

**FIGURE 5:**
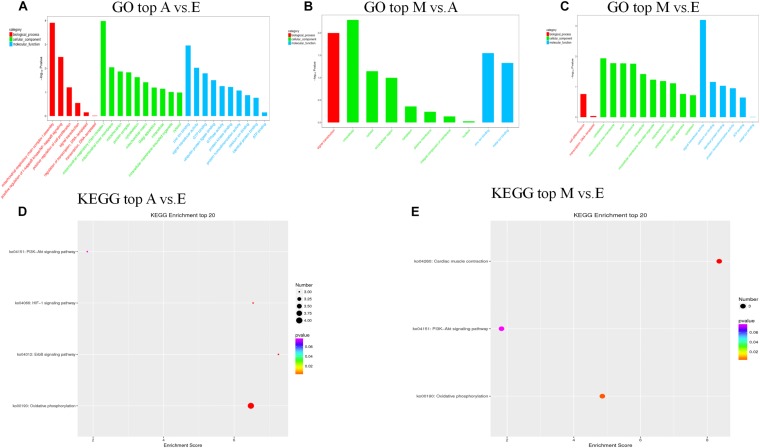
The top GO and KEGG enrichment analyses of the trans-target genes of the differentially expressed lncRNAs. **(A–C)** The top GO terms enriched by the target genes of the differentially expressed lncRNAs in each comparison. **(D,E)** The top KEGG terms enriched by differentially expressed lncRNAs in each comparison (A–E, M–E).

We further predicted potential target genes of lncRNAs involved in trans regulation based on Pearson’s correlation coefficients (|*r*| > 0.95). In the A vs. E group, 21 lncRNAs (15 annotated lncRNAs and six novel lncRNAs) corresponded to 82 target genes. DEIncRNA target genes were enriched in 38 GO functions, of which only 12 were significantly enriched (*P* < 0.05). The results showed that these lncRNA targets were enriched in multiple processes, such as mitochondrial respiratory chain complex I, mitochondrial respiratory chain complex I assembly, mitochondrial matrix, and cytoskeleton. KEGG analysis of these DElncRNAs indicated that target genes were involved in oxidative phosphorylation, ErbB signaling pathway, HIF-1 signaling pathway, and PI3K-Akt signaling pathway (Figures 5A,D and Supplementary Table S6). In the M vs. A group, nine lncRNAs (eight annotated lncRNAs and one novel lncRNAs) corresponded to 35 target genes. DEIncRNA target genes were enriched in 10 GO functions of which only four were significantly enriched (*P* < 0.05). The trans-regulatory target genes in the M. vs A group were not enriched in any KEGG pathway, and the results were consistent with the previous cis target gene enrichment, which may be caused by the low number of target genes in the M. vs A group (Figure 5B and Supplementary Table S6). In the M vs. E group, 26 lncRNAs (22 annotated lncRNAs and four novel lncRNAs) corresponded to 86 target genes. DEIncRNA target genes were enriched in 28 GO functions, of which only six were significantly enriched (*P* < 0.05) in categories such as signal transducer activity, cytoskeleton, and mitochondrial inner membrane. KEGG analysis of these DElncRNAs indicated that target genes were involved in cardiac muscle contraction, oxidative phosphorylation, and PI3K-Akt signaling pathway (Figures 5C,E and Supplementary Table S6).

### LncRNA-mRNA Co-Expression Network Analysis

**FIGURE 6:**
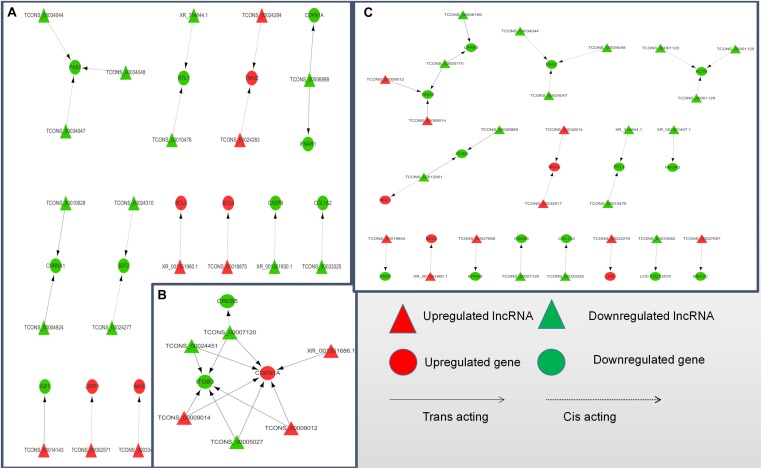
LncRNA-target gene networks in (control/experiment) A/E, M/A, and M/E comparisons. **(A)** LncRNA-gene network for the A/E comparison. **(B)** LncRNA-gene network for the M/A comparison. **(C)** LncRNA-gene network for the M/E comparison. The network of differentially expressed lncRNAs and the corresponding cis and trans regulated target genes is shown. In this network, red represents upregulation, green represents downregulation, triangles represent lncRNA, circles represent mRNA, dotted lines represent cis regulation, and solid lines represent trans regulation.

**FIGURE 7:**
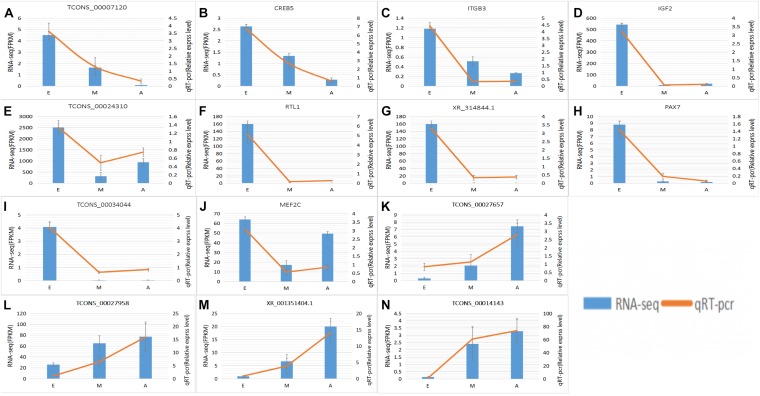
Expression levels of differentially expressed IncRNAs and the co-expressed target genes and validated by qRT-PCR. **(A–N)** RNA-Seq results are shown as a bar graph. The value to the right of the *Y*-axis represents FPKM. The qRT-PCR results are shown in the line graph, with the *Y*-axis on the left representing the relative expression level. Data represent mean ± SE.

To further explore how lncRNA and target genes regulate muscle growth, we performed co-expression analysis of the target genes of the DElncRNAs based on lncRNA FPKM. A total of 19 (A to E), 25 (M to A), and six (M to E) lncRNA gene pairs were obtained for each. These three networks provided candidate lncRNAs associated with muscle growth (Figure 6). Consequently, two biological processes identified in the GO analysis (*in utero* embryonic development and muscle organ development) and five KEGG pathways related to muscle development (PI3K-Akt signaling pathway, focal adhesion, MAPK signaling pathway, apoptosis, and p53 signaling pathway) corresponding with 27 mRNAs and 39 lncRNAs were obtained, including retrotransposon-like 1 (RTL1) and insulin-like growth factor 2 (IGF2) (Supplementary Table S7). The qRT-PCR analysis showed that, in the process of muscle growth, the expression of lncRNA corresponded with changes in target genes, including CREB5, ITGB3, TCONS_00007120, IGF2, TCONS_00024310, RTL1, XR_314844.1, PAX7, TCONS_00034044, MEF2C and TCONS_00027657. TCONS_00027657, TCONS_00014143, XR_001351404.1, and TCONS_00027958 are increased expression lncRNAs during development (Figure 7). These results were consistent with the RNA-Seq data, which confirmed the reliability of the sequencing data (*R* = 0.98, *P* < 2.2e-16) (Supplementary Figure S1). These results further revealed the identity and relationships among target genes of the lncRNA. However, further studies are required to fully elucidate the lncRNA sequences and loci.

## Discussion

In recent years, the development of high-throughput sequencing technology and new molecular biology techniques has greatly promoted the study of skeletal muscle lncRNAs (Wang et al., 2019), and accumulating evidence indicates that lncRNA is in mouse (Butchart et al., 2016), pig (Zhao et al., 2015), cattle (Sun et al., 2016), goat (Zhan et al., 2016), and human (Iyer et al., 2015) play important roles in muscle development. Compared with the lncRNAs identified in other model organisms, the identification and characterization of yak lncRNAs (especially those associated with skeletal muscle development) is very limited. In our study, we used high-throughput sequencing and bioinformatics analysis at three time-points to systematically identify yak lncRNAs in LD muscle for the first time.

Non-coding and protein-coding genes are known for their potential coding ability. In this study, we utilized highly stringent filtration pipeline (Figure 1) to minimize interference by false positive results and to remove transcripts with evidence of protein-coding potential. We identified 1180 lncRNAs at three developmental stages of skeletal muscle, with high confidence. Investigations of other organisms have revealed similarities in the number of exons and length of the identified lncRNA exons (Cabili et al., 2011; Pauli et al., 2012). LncRNAs have fewer exons, shorter transcription lengths, and lower expression levels than mRNA (Figures 2C,D). In this study to detect the presumption of lncRNA number in the two reports about the research between the number of yak muscle lncRNA (Billerey et al., 2014; Sun et al., 2016).

LncRNAs are endogenous non-protein-encoding transcripts that function as regulators of gene expression and participate in biological development processes (Mercer et al., 2008; Knoll et al., 2015). In this study, we identified trends in the expression of lncRNAs in the three stages of yak development. Some lncRNAs were found to be strongly expressed during the fetal phase, while others were more prominent in infancy and adulthood. The qRT-PCR results further confirmed that the DElncRNA and DEG expression were consistent with the sequencing data. We searched for target genes of lncRNA in cis- and trans-acting processes and functional annotation based on the obtained target genes to reveal the role of the lncRNAs. GO analysis of the target genes of DElncRNAs indicated that the DElncRNAs identified in the in the comparison of the A vs. E and M vs. E groups are involved in several important biological functions and processes, such as *in utero* embryonic development (CUL4A; IGF2; TANC2; IFITM5; and RIC8A) and muscle organ development (ANKRD2; MEF2C; and MKL2). However, GO entries not directly related to muscles were not found in the M vs. A group, probably due to incomplete yak reference genomes. In addition, the IGF2 gene and co-expressed lncRNAs (TCONS_00024277 and TCONS_00024310) are the most worthy of attention since this is a major regulator of muscle development and growth (Wilson et al., 2003). Previous studies have shown that IGF2 belongs to the imprinted H19-IGF2 locus. H19 regulates IGF2 expression during muscle differentiation (Borensztein et al., 2013). H19 was one of the first lncRNAs to be studies and plays wide-ranging roles in organisms, participating in the regulation of the growth and development of mammalian embryonic tissues by acting in the cis and trans modes on target genes (Qin et al., 2017). Zhu et al. (2017) identified a myogenesis-associated lncRNA (lnc-mg) that promotes skeletal muscle stem cell differentiation and muscle regeneration by acting as a competing endogenous RNAs (ceRNAs) that competitively bindings to miR-125b and enhances IGF2. This interaction activates downstream signaling pathways for skeletal muscle stem cell differentiation and regeneration, thereby promoting muscle production. These studies demonstrate that this lncRNA plays an important role in muscle development as an endogenous competitive RNA. In this study, we found that two lncRNAs mediated cis regulation of IGF2 as the target gene. In the A vs. E group comparison, the expression of two lncRNAs was similar to that of IGF2, which was higher in the fetal group than in the adult group. Therefore, we speculated that these two lncRNAs play regulatory roles in the development of fetal yak calf muscles, although further experiments are needed to verify the function of these lncRNAs and to analyze the predicted target genes. Recent advances in skeletal muscle research have highlighted the close relationship between some of the key signaling pathways and skeletal muscle growth and development (Mukund and Subramaniam, 2020). Our KEGG analysis confirmed that several DElncRNAs identified in the A vs. E, M vs. A and M vs. E group comparisons might be related to muscle biology, particularly the PI3K-Akt signaling pathways. The PI3K-Akt signaling pathway plays a crucial role in the synthesis and degradation of proteins in skeletal muscle, which also plays a role in resisting muscle atrophy (Schiaffino et al., 2013; Zhang et al., 2016). As the species of yak used in this study inhabit a plateau hypoxic environment, two other pathways of concern (MAPK and HIF-1 signaling pathway) were found in the KEGG enrichment analysis. Previous studies showed that under hypoxic conditions, PI3K activation can be achieved through activation of these pathways to inhibit apoptosis and proteolysis, and reduce the damage caused by hypoxia (Ding et al., 2017). Activation of the PI3K-Akt (BelAiba et al., 2007) and ERK1/2MAPK (Agani and Jiang, 2013) signaling pathways can protect muscle cells from oxidative damage and apoptosis. To adapt to the hypoxic environment, mammalian cells actively promote the expression of hypoxia-induced genes such as vascular endothelial growth factor (VEGF) as well as various glycolytic enzymes and glucose transporters. The expression of these genes is affected by HIF-1 regulation. Studies have shown that the expression and activity of HIF-1α is affected by the extracellular signal-regulated kinase (ERK) pathway. Acute hypoxia can increase myocardial central atrial peptide (ANP) secretion and HIF-1α transcription and expression levels. Hypoxia also activates MAPK and PI3K signaling pathways to regulate HIF-1α activity in the heart (Zhang et al., 2013). Therefore, based on these results, we speculate that both comparison groups were found in the fetal calf and the two groups after birth (M vs. E and A vs. E), but the same results were not found in the M vs. A comparison group. To adapt to the external plateau environment after birth, it can be speculated that the PI3K-Akt, MAPK, and HIF-1 pathways are activated in yak to protect the muscle cells from oxidative damage and apoptosis after birth.

According to the critical path of muscle development and validation of a number of previously identified key genes, as well as the DElncRNAs and their target genes identified in this study, we constructed an lncRNA-mRNA interaction to better demonstrate their co-regulatory relationships. According to the network, some target gene has been reported to be a muscle growth regulator, such as RTL1, and two lncRNAs (XR_314844.1 and TCONS_00010478) were identified as cis-acting regulated of RTL1 and simultaneously appeared in the A vs. E and M vs. E comparison groups. The RTL1 gene, also known as PEG1I (paternally expressed gene11), is a Sushi-like retrotransposon that loses its ability to autonomically encode a protein homologous to Gag and Pol proteins (Youngson et al., 2005). This gene is and located in the imprinted gene locus of DLK1 and type III iodothyroid hormone deiodiase genes (dlk1-dio3) and was reported be to highly expressed in embryonic and placental tissues, playing an important role in maternal placenta and fetal nutrient delivery. Deletion or overexpression of RTL1 in mice leads to retardation of fetal growth or neonatal development (Sekita et al., 2008). RTL1 was also found to be associated with muscle hypertrophy in sheep (Fleming-Waddell et al., 2009). In our study, we observed higher expression of RTL1 genes and two co-expressed lncRNAs in the fetal yak group compared with the levels at the other two development stages, indicating that the regulatory pattern of this lncRNA plays a key role in the embryonic phase of yak development. In addition to RTL1-related lncRNA, the role of TCONS_00027657 (identified in the M vs. E comparison) in the regulation of MEF2C (myocyte enhancer factor 2C) was also brought to our attention. MEF2C is a member of the MEF2 family, which regulates the proliferation and differentiation of various cells. The MEF2C gene is tissue-specific and is present in muscle tissue, especially skeletal muscle and myocardium (Fickett, 1996). MEF2C binds directly to some muscle-specific gene promoters to regulate muscle development. In MEF2C gene knockout mice, muscle fibers rapidly develop lesions after birth, which leads to disorders of myocardial, skeletal, and striated muscle development (Potthoff et al., 2007). Furthermore, Juszczuk-Kubiak et al. (2011) showed that MEF2C can be used as a genetic marker of bovine corpus callosum and meat quality traits. In mouse and human myoblasts, linc-MD1 (long intergenic non-coding RNA muscle differentiation 1) binds competitively to miR-133 and miR-135, thereby activating the target genes MAML1 (metastasis-associated lung adenocarcinoma transcript 1) and MEF2C, respectively, and accelerating muscle transition to a late differentiation stage (Cesana et al., 2011). In summary, the MEF2C gene plays a crucial role in muscle development.

The DElncRNAs TCONS_00034044, TCONS_00034047, and TCONS_00034048 identified in the A vs. E and M vs. A networks were predicted to target Pax7, which plays an important role in the early stage of embryonic development, neural tube formation, neural crest development, and paraxial mesoderm development (Cai et al., 1995). This gene has been has been shown to promote the development of young mice (Jostes et al., 1990), and the fish central nervous system (Jostes et al., 1990), with mutations causing serious organ development defects (Thomas et al., 2007). It also plays an important role in the repair of adult optic nerve injury, skeletal muscle development and self-renewal, and regeneration and repair of muscle damage in human (Hyatt et al., 2008) and mouse (Jostes et al., 1990). In this study, we showed that the expression of Pax7 in fetal yak muscle was higher than that in the other two groups, with no difference in expression levels between the other two groups, which is consistent with previous reports of Pax7 expression in the longissimus dorsi of different age groups in pigs (Patruno et al., 2008).

## Conclusion

This study is the first to provide a systematic description of lncRNAs at different stages of yak muscle development. A series of lncRNAs and target genes related to yak muscle development were screened by Illumina sequencing platform. The lncRNAs identified in this study have a number of characteristics similar to those of other mammals. With reference to GO and the KEGG database, we showed that the target genes of the DElncRNAs were involved in multiple biological processes associated with muscle. Furthermore, the visual representation of the candidate lncRNA gene transcription regulation network provides a valuable resource for exploration of the function of the yak lncRNAs. In future studies, we plan to investigate the function of these lncRNAs to gain a comprehensive understanding of the molecular mechanism underlying yak muscle development.

## Data Availability Statement

Publicly available datasets were analyzed in this study. These data can be found here: https://dataview.ncbi.nlm.nih.gov/object/PRJNA550017.

## Ethics Statement

The animal study was reviewed and approved by the Animal Administration and Ethics Committee of Lanzhou Institute of Husbandry and Pharmaceutical Sciences of Chinese Academy of Agricultural Sciences (Permit No. 2019-002). All yaks were handled in strict accordance with good animal practices by following the Animal Ethics Procedures and Guidelines of the People’s Republic of China. Written informed consent was obtained from the owners for the participation of their animals in this study.

## Author Contributions

PY and CL conceived and designed the experiments and explained the data. XM analyzed the main content of the data with the help of MC, XD, XW, XG, JP, and PB. DF performed the experiments with the help of QK. XM wrote the manuscript with the help of PY and CL.

## Conflict of Interest

The authors declare that the research was conducted in the absence of any commercial or financial relationships that could be construed as a potential conflict of interest.

## Footnotes

1 https://github.com/alexploner/Heatplus

2 www.kegg.jp/kegg/kegg1.html

## Supplementary Material

FIGURE S1 Correlation between the qRT-PCR and the RNA-seq data. The trend lines and formula in each scatter plot represent the correlation coefficients.

TABLE S1 Primers for qRT-PCR.

TABLE S2 Summary of RNA-seq data and reads mapped to the *Bos mutus* reference genome.

TABLE S3 Novel lncRNA classification.

TABLE S4 List of differentially expressed lncRNAs from four skeletal muscle developmental stages.

TABLE S5 Cis-target gene enrichment GO and KEGG analysis of each group.

TABLE S6 Trans-target gene enrichment GO and KEGG analysis of each group.

TABLE S7 Muscle growth-associated differentially expressed lncRNAs and their target genes in each group.
