# Supplementary figures and images for: Genome-Wide Analysis Reveals Changes in Polled Yak Long Non-coding RNAs in Skeletal Muscle Development
Source: Front Genet. 2020 Apr 15;11:365. doi: 10.3389/fgene.2020.00365 (PMC7176074; doi:10.3389/fgene.2020.00365)

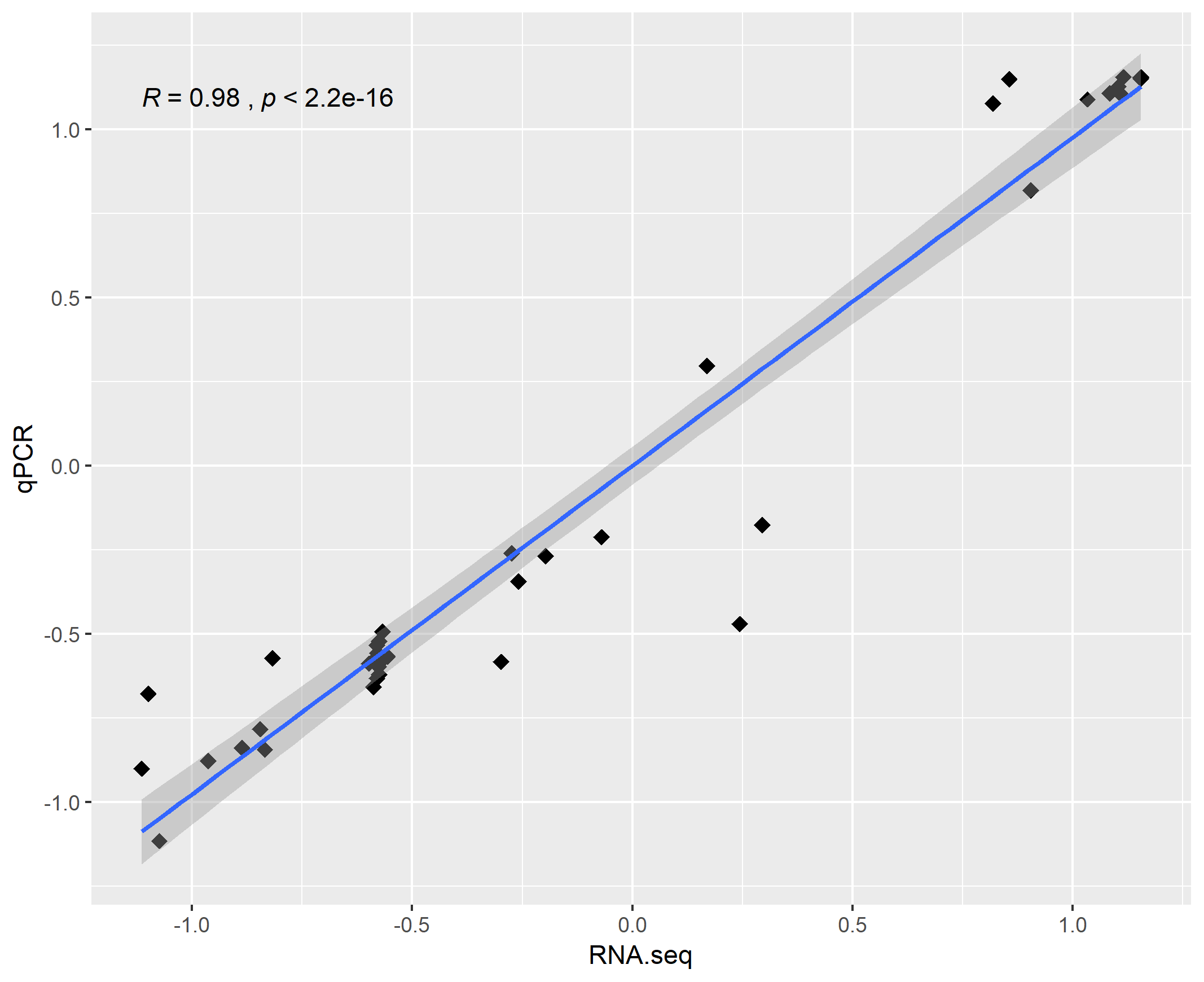

Supplement: FIGURE S1 — Correlation between the qRT-PCR and the RNA-seq data. The trend lines and formula in each scatter plot represent the correlation coefficients. [file Image_1.TIFF]
